# Monitoring eruption activity using temporal stress changes at Mount Ontake volcano

**DOI:** 10.1038/ncomms10797

**Published:** 2016-02-19

**Authors:** Toshiko Terakawa, Aitaro Kato, Yoshiko Yamanaka, Yuta Maeda, Shinichiro Horikawa, Kenjiro Matsuhiro, Takashi Okuda

**Affiliations:** 1 Earthquake and Volcano Research Center, Graduate School of Environmental Studies, Nagoya University, D2-2 (510) Furo-cho Chikusa-ku, Nagoya 464-8601, Japan

## Abstract

Volcanic activity is often accompanied by many small earthquakes. Earthquake focal mechanisms represent the fault orientation and slip direction, which are influenced by the stress field. Focal mechanisms of volcano-tectonic earthquakes provide information on the state of volcanoes via stresses. Here we demonstrate that quantitative evaluation of temporal stress changes beneath Mt. Ontake, Japan, using the misfit angles of focal mechanism solutions to the regional stress field, is effective for eruption monitoring. The moving average of misfit angles indicates that during the precursory period the local stress field beneath Mt. Ontake was deviated from the regional stress field, presumably by stress perturbations caused by the inflation of magmatic/hydrothermal fluids, which was removed immediately after the expulsion of volcanic ejecta. The deviation of the local stress field can be an indicator of increases in volcanic activity. The proposed method may contribute to the mitigation of volcanic hazards.

## Abstract (web summary)

Volcanic activity is often accompanied by small earthquakes, with focal mechanisms indicating stress state beneath volcanoes. Here, the authors demonstrate that quantitative evaluation of temporal stress changes is an effective tool for eruption monitoring.

## Graphical abstract

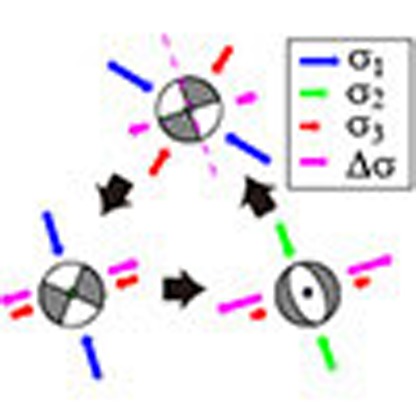

The local stress field around volcanoes represents the superposition of the regional stress field and stress perturbations related to volcanic activity^1^,^2^,^3^,^4^. Temporal stress changes over periods of weeks to months are generally attributed to volcanic processes. Examining the focal mechanism solutions of volcano-tectonic (VT) earthquakes provides detailed information on the state of a volcano via the local stress field. This has the potential to contribute to prediction of eruptions over the short and medium term (weeks to months).

Mt. Ontake volcano is the second highest stratovolcano in Japan and is located at the southern end of the Northern Japan Alps. On 27 September 2014, around 11:52 hours JST (UTC+9), Mt. Ontake produced a hydrothermal (steam-type) eruption with a volcanic explosivity index (VEI) value of 2 (ref. 5). Mt. Ontake had been considered dormant until its first historical eruption in October 1979. The 2014 eruption struck on a sunny Saturday in the autumn foliage season, resulting in the worst volcanic disaster in Japan in the last 90 years: 58 people died and 5 are still missing.

Mt. Ontake is continuously monitored by several relatively dense networks of permanent seismic stations operated by Nagoya University, Japan Meteorological Agency (JMA), National Research Institute for Earth Science and Disaster Prevention (NIED), and Gifu and Nagano prefectures (Fig. 1a,b). The summit region of Mt. Ontake (Fig. 1a,b; latitude 35.875° N–35.900° N, longitude 137.44° E–137.50° E) is normally almost aseismic, with an average of less than one event (*M*<0) per month, whereas the surrounding region is characterized by a high level of background seismicity (see Methods). The onset of VT earthquakes was observed on 31 August 2014 (Fig. 1b,c), reaching a peak on 11 September 2014 before decaying over time until the eruption, although the seismicity increased again between 15 and 21 September 2014 during this period. Long-period earthquakes with peak frequencies of 1–5 Hz were recorded beginning around 15 September, but less than 10 long-period events were detected in real time. Application of a matched-filter technique to continuous waveforms (23 August to 30 September 2014) resulted in detection of thousands of micro-seismic events, indicating that an increase in VT events was followed by an increase in the number of long-period events 5 days later^6^. This detailed analysis also revealed that VT events beneath the craters migrated upwards as well as laterally in the NNW–SSE direction for the final 10 min preceding the eruption^6^. The phenomenon synchronized with a rapid increase of pre-eruptive tremor amplitudes and with an unusual tiltmeter signal indicating summit upheaval^6^. After the eruption, the magnitudes of events became larger (*M*<1); in contrast, most pre-eruption events had a magnitude of less than 0.

The time history of volcanic earthquakes during the pre-eruption period was explained by the generic volcanic earthquake swarm model^7^. In this respect, the history was similar to that associated with a previous minor eruption in 2007 (VEI=0)^8^. However, unlike the 2007 eruption, the 2014 eruption was not preceded by active volcanic tremors and inflation of the volcanic edifice until 7 min before the eruption^6^,^9^. The time scale for the precursor period was also different from that in 2007. In general, the various observations of precursor processes make eruption forecasting difficult, although changes in volcanic seismicity have been successfully used empirically for predicting eruptions^10^,^11^.

The local stress field beneath volcanoes is directly influenced by the inflation of the ascending magma and magmatic/hydrothermal fluids^1^,^2^,^3^,^4^. As the focal mechanism solution of VT events contains information on the local stress field, the relationships between stresses and volcanic processes have been studied by examining focal mechanisms and using theoretical models with the aim to successfully predict eruptions^1^,^2^,^3^,^4^,^12^,^13^,^14^,^15^,^16^,^17^. However, systematic spatiotemporal changes in focal mechanisms do not necessarily indicate changes in the stress field^18^, because earthquakes can be triggered at faults that are misoriented to the stress field by a decrease in fault strength caused by an increase in pore fluid pressure^19^,^20^. This effect cannot be ignored when evaluating the local stress fields beneath active volcanoes because the existence of over-pressurized fluids is certain.

Here we propose a method to detect true temporal changes in the local stress field beneath Mt. Ontake by examining the focal mechanism solutions of VT events relative to the regional stress pattern. Enhanced volcanic activity causes significant stress perturbation with E–W tension, using the crustal structure controlled by the regional stress field. This results in a large deviation of the local stress field from the regional stress field before the 2014 eruption. We demonstrate that quantitative evaluation of temporal stress changes is an effective tool for eruption monitoring.

## Results

### Focal mechanism solutions of VT earthquakes

**Figure 1:**
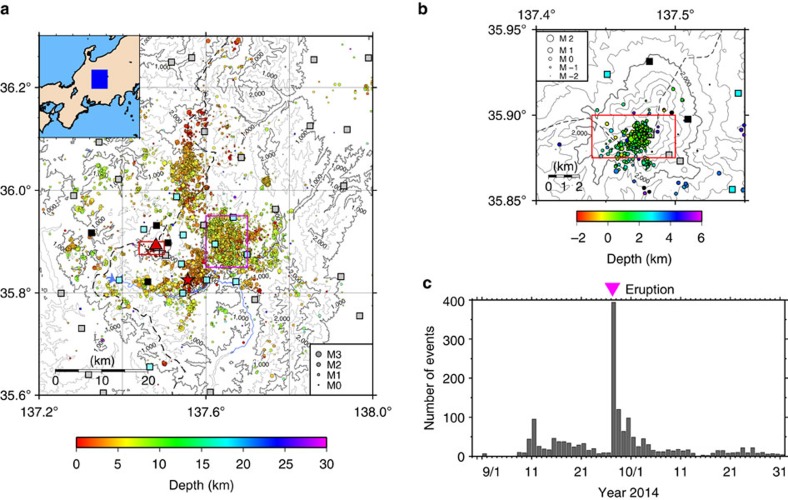
Seismicity around Mount Ontake. (**a**) Earthquake hypocentres and seismic stations in the Ontake region and (**b**) summit region. The circles denote hypocentres, and the colour scale corresponds to focal depth. The light blue, grey and black squares denote seismic stations operated by the Nagoya University, the national institutes and prefectural institutes, respectively. The blue rectangle in the inset in **a** shows the location of the Ontake region. The triangle, star, red rectangles and pink rectangle denote Mt. Ontake, the hypocentre of the 1984 Western Nagano Prefecture Earthquake, the summit region and the region depicted in Fig. 3b, respectively. The black dashed lines in **a**,**b** indicate the boundary between Nagano and Gifu prefectures. (**c**) Histogram of VT events per day in the summit region.

**Figure 2:**
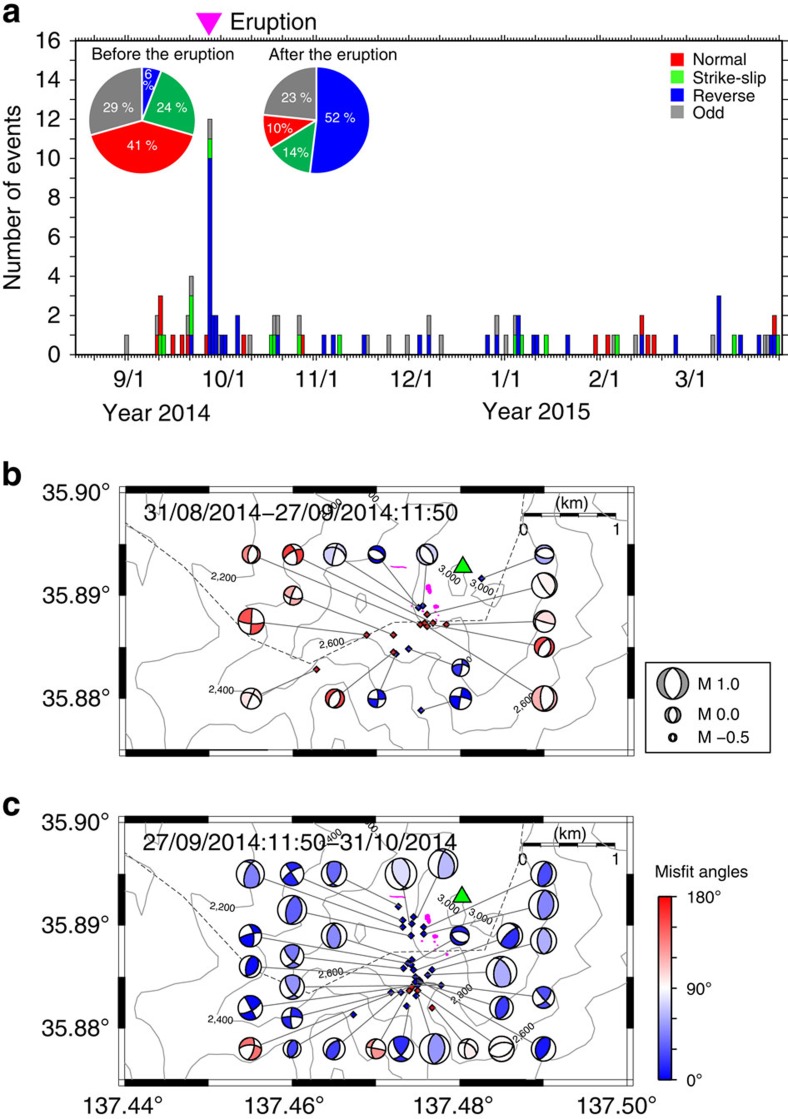
Temporal changes in focal mechanism solutions. (**a**) Classification of focal mechanism solutions^22^. The pie charts show the proportions of the four faulting types before and after the eruption. (**b**) Focal mechanism solutions for the precursory period. (**c**) Focal mechanism solutions for the first post-eruption period (27 September 2014 to 31 October 2014). Solutions are represented by lower hemisphere projections of focal spheres. The diamonds denote event epicentres. The colour scale indicates misfit angles. Pink spots mark the craters of the 2014 eruption^33^.

We estimated the focal mechanism solutions of 94 VT earthquakes in the summit region (August 2014 to March 2015) from S:P amplitude ratios and P wave polarity data obtained through the dense seismic networks (Fig. 1a) using the HASH software package^21^. In the analysis, we assumed that the source was double couple (DC) and considered three velocity models (Supplementary Fig. 1) to account for possible errors in the hypocentres and take-off angles (see Methods). These solutions (Supplementary Table 1) were classified into four types based on the angle of the pressure, null and tension axes (P, N and T axes, respectively) with respect to the horizontal^22^: normal faulting, strike–slip faulting, reverse faulting and others (odd faulting) (Fig. 2a). The pre-eruption seismicity was dominated by normal faulting, which accounted for 7 (=41%) of the 17 events in this period. The mean orientation of the T axes during the precursory period was N75° E with an s.d. of 13° (Fig. 2b and Supplementary Fig. 2a). After the eruption, in contrast, 40 (=52%) of the 77 events showed reverse faulting, and normal faulting events became relatively rare (Fig. 2a). The mean orientation of the P axes during the post-eruption period was N101° E with an s.d. of 13° (Fig. 2c and Supplementary Fig. 2b), sub-parallel to the T axes of the precursory period, indicating that the local stress field was markedly different during the periods before and after the eruption.

### Regional stress field around Mt. Ontake

To determine the relationships of the focal mechanism solutions to the regional stress field, we estimated the regional stress pattern around Mt. Ontake from 536 focal mechanism solutions (*M*≥1) during a period of typical background seismicity (May 2012 to July 2014) using the centroid moment tensor (CMT) data inversion method^23^ (see Methods). The stress field was roughly characterized by strike–slip faulting in the entire region. The axes of the maximum and minimum compressive principal stresses were sub-horizontal in the summit region, oriented N64° W and N25° E, respectively (Supplementary Fig. 3), consistent with the previous studies^24^,^25^,^26^. The orientations of the maximum and minimum horizontal principal stress axes varied within N57° W–N72° W and N17° E–N33° E, respectively (see Methods). In a different region with an active micro-seismic swarm (Fig. 1a; latitude 35.85° N–35.95° N, longitude 137.6° E–137.7° E) the stress pattern was very similar to that in the summit region, but the fluctuation ranges of the principal stress axes were smaller (<10°) because of sufficient data for the inversion.

### Temporal evolution of misfit angles

We calculated the misfit angle *φ* of focal mechanisms between the observed slip vectors and theoretical slip vectors expected from the regional stress field on the basis of the concept that seismic slip occurs in the direction of the resolved shear traction acting on a pre-existing fault^27^,^28^ (see Methods). When the misfit angles are less than the estimation errors of the regional stress field and focal mechanism solutions, events are considered to be consistent with the regional stress field, because the actual slip vector agrees with the theoretical one within the estimation errors. For events in the summit region, the threshold value was ∼65° (errors in stresses: <20°; errors in focal mechanisms: <45°). Focal mechanism solutions with *φ*≥90° are considered highly inconsistent because the actual and theoretical slip vectors are opposite. Before the eruption, only 5 (=29%) of the 17 VT events were consistent with the regional stress field and 10 (=59%) of the events were highly inconsistent (Fig. 2b and Supplementary Fig. 4). However, the ratio of consistent events increased to >70% during the period between the eruption and the end of December 2014 (Fig. 2c and Supplementary Fig. 4).

**Figure 3:**
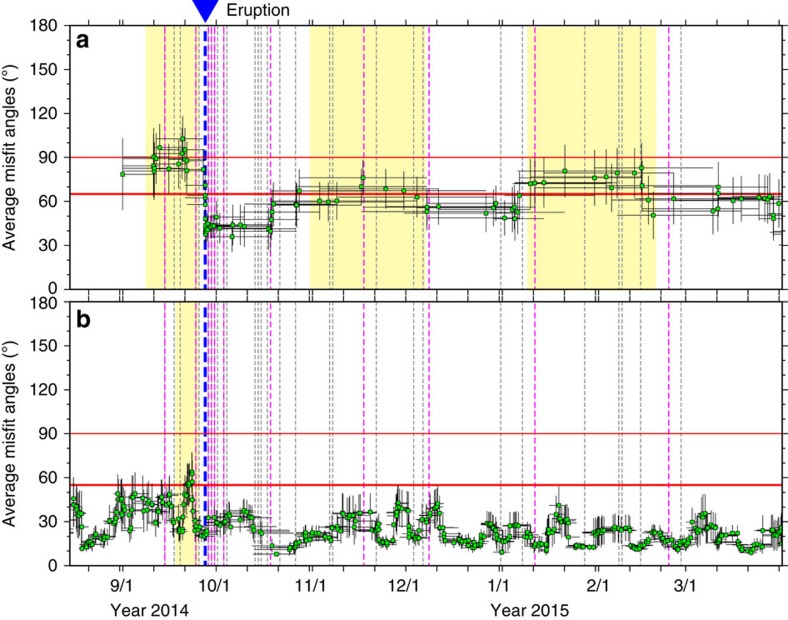
Temporal evolution of average misfit angles. The moving average of misfit angles of focal mechanism solutions in the summit region is shown in **a** and that in the peripheral swarm region (Fig. 1a) is illustrated in **b**. The light green squares in both panels denote average misfit angles. The black horizontal bars denote the time window for calculating average misfit angles, and the black vertical bars denote the s.e. The blue, pink and grey dotted lines show the origin times of the 2014 eruption, low-frequency B-type (BL) events and high-frequency B-type (BH-type) events estimated by JMA. The thick red lines show the threshold angles (65° and 55°) to detect deviations of the local stress field in the summit and peripheral swarm regions, respectively, (Fig. 1a) from the regional stress field. The thin red lines show another threshold angle (90°) to detect a large inconsistency of the local stress field to the regional stress field. The yellow hatched zones indicate enhancements of misfit angles above the threshold values.

We calculated the moving average of misfit angles over a varying time window containing 10 events, shifting the window by 1 event per calculation (Fig. 3a). The average misfit angle significantly exceeded the threshold value (65°) prior to the eruption. Immediately after the eruption, however, the average misfit angle showed a marked decrease. A less pronounced enhancement of the quantity was also observed during January to February 2015, followed by a gradual decrease at the end of February 2015. Meanwhile, such remarkable temporal changes in average misfit angles were not observed in the peripheral swarm region (Fig. 1a), in which 89% of events had misfit angles less than a threshold value of 55° (errors in stresses: <10°; errors in focal mechanisms: <45°), consistent with the regional stress field during the entire period (Fig. 3b).

## Discussion

The velocity structure beneath Mt. Ontake is not well-known. Small changes in the hypocentre depends on variations in the velocity structure, which may produce large variations in take-off angles, making stable estimation of focal mechanism solutions difficult^12^,^29^. For events in the summit region, epicentral locations were basically independent of the velocity model (Supplementary Fig. 1) in which the differences between the three models were 130 m on average, but the dependency of hypocentral depths was not negligible. The hypocentral depths with Model 1 were 700 m shallower on average than those with Model 2 and 1 km deeper than those with Model 3. To examine the effects of uncertainty of the velocity model, we estimated focal mechanism solutions for each of the three velocity models and calculated the moving average of misfit angles for each case (Supplementary Fig. 5). In all cases, the three remarkable characteristics obtained from the stable data set of focal mechanism solutions (Fig. 3a) were commonly reproduced. This finding strongly indicates that the analysis of misfit angles was robust and insensitive to the assumed velocity structures.

A significant deviation of the local stress field was expected to have occurred during the pre-eruption period. The most plausible physical mechanism for this change is a significant stress perturbation due to enhanced volcanic activity^1^,^2^,^3^,^4^,^15^,^16^,^30^,^31^,^32^. It is not unusual for the slip direction of post-eruption events to be controlled by the regional stress field because stress perturbations induced by movement of magmatic/hydrothermal fluids may be negligible^32^. The many reverse faulting events contributed to shrinkage of the volcanic edifice after expulsion of volcanic ejecta.

**Figure 4:**
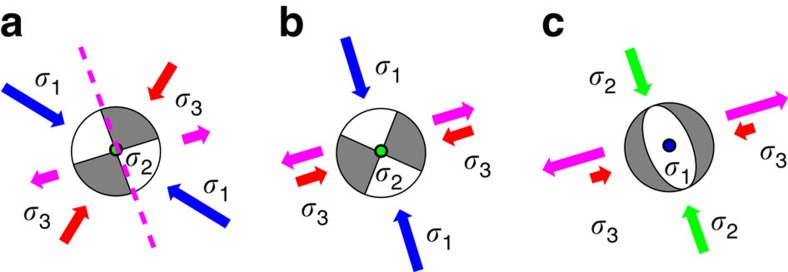
Stress field rotation caused by volcanic activity. (**a**) The regional stress pattern in the summit region. (**b**) Horizontal stress rotation. (**c**) Vertical stress rotation. The stress pattern is shown by the focal spheres (lower hemisphere projections) in which nodal planes are the maximum shear planes. The blue, light green and red arrows show the axes of the maximum (*σ*_1_,), intermediate (*σ*_2_) and minimum (*σ*_3_) compressive principal stresses, respectively. The pink dashed line in **a** indicates the strike of possible inflating cracks. The pink arrows mark the main direction of crack opening.

The mean orientation of the T axes of pre-eruption events (N75°E±13°) was roughly perpendicular to the near-vertical plane (strike N15 °W) on which the relocated hypocentres of VT events were concentrated^6^ and to the alignment of craters from the 2014 eruption (Fig. 2b,c)^33^. Prior to the 2007 and 2014 eruptions, very long-period earthquakes with a period of >20 s were recorded at broadband seismic stations: the sources of these earthquakes are modelled by tensile cracks with strikes of N20° W (for the 2007 event)^8^ and N6° W (for the 2014 event)^9^. These results suggest that a volcanic system exists beneath Mt. Ontake in which inflation is driven by magmatic/hydrothermal fluids propagating upwards in a vertical crack. We suggest that the inflation caused a stress perturbation with E–W to ENE–WSW tension, which altered the local stress field during the period leading up to the eruption (Fig. 4).

The strike of this possible conduit for magmatic/hydrothermal fluids is sub-parallel to that of a maximum shear plane of the regional stress field (Fig. 4a and Supplementary Fig. 3), indicating that the Mt. Ontake eruption conduit followed structures with orientations controlled by the regional stress field^32^,^34^. When the inflationary pressure is sufficiently high, clockwise rotation of the maximum and minimum principal stress axes occurs ahead of a propagating tensile crack (Fig. 4b)^35^. Strong tension may weaken the principal horizontal compressive stresses, meaning that the (vertical) intermediate principal stress becomes the maximum stress, resulting in a local stress field characterized by normal faulting with E–W tension (Fig. 4c). An increase in *b*-values during the pre-eruption period, although the values gradually declined from 16 September 2014 to just before the eruption, may be related to strengthening of the tensional stress field due to the enhanced hydrothermal activity^6^,^36^.

As the hypocentral depths during the post-eruption period were systematically <1 km shallower than those before the eruption^6^, we cannot rule out that the regional stress field in the summit region is heterogeneous on a small scale. If the regional stress field was locally characterized by E–W tension in the source region of the precursory events, stress perturbation with E–W tension would increase the deviatoric stress, which would promote seismic slip with positive changes in the Coulomb failure function^37^. As the summit region is usually aseismic, activation of normal faulting with E–W tension can be an indicator of an increase in volcanic activity.

The time history of average misfit angles showed another slight enhancement in November 2014 other than that in January–February 2015. This indicates that re-pressurization of magmatic/hydrothermal fluids beneath Mt. Ontake commenced again at the beginning of October 2014. The decreases in misfit angles following the two enhancements indicate that some de-pressurization of magmatic/hydrothermal fluids, for example, as a result of an undetected minor eruption, occurred at the end of November 2014 and February 2015.

The local stress field in the peripheral swarm region (Fig. 1a) slightly deviated from the regional stress field for a week before the 2014 eruption. An inversion analysis of focal mechanism solutions, repeated precise levelling measurements, magnetotelluric measurements, and geochemical analyses of water and gas samples demonstrated the presence of over-pressurized fluids in the region^38^,^39^,^40^,^41^. The porous network in the underground rock around Mt. Ontake is not well-known^42^. However, pore fluid pressures in the peripheral swarm region may have been increased by the volcanic processes at Mt. Ontake, which in turn may have caused temporal changes in the stress field of the peripheral swarm region. In that case, interaction between the volcanic eruption and inland earthquakes would be important. Five years after the first historic eruption of Mt. Ontake in 1979, the Western Nagano Prefecture earthquake (Mj 6.8) occurred in the southeast flank of the volcano in 1984 (Fig. 1a): the two events may have been linked.

Spatiotemporal changes in the local stress field have been reported at many volcanoes through stress inversion and/or by examining the P and T axes of focal mechanism solutions^12^,^13^,^14^,^15^,^16^. However, focal mechanisms are controlled by not only stresses but also fault strength, which is influenced by pore fluid pressures^19^,^20^,^38^,^43^,^44^. As earthquakes release stresses on pre-existing faults by shear faulting, a decrease in fault strength due to an increase in pore fluid pressures can trigger events on faults that are misoriented to the regional stress field^19^,^20^,^44^. From a data set of biased focal mechanism solutions by over-pressurized fluids, we may overestimate changes in the stress field or yield apparent stress rotation^20^ because the method of stress inversion attributes spatiotemporal variation in focal mechanisms only to those stresses. The method of shear-wave splitting analysis is also useful to detect temporal changes in the local stress field^30^,^31^, but a similar problem with apparent stress rotation has been reported^45^. In contrast, the average misfit angle of focal mechanism solutions in the context of regional stress patterns is crucial in detecting true temporal changes in local stress fields. However, we may underestimate temporal changes in stresses because the method attributes variation in focal mechanisms to changes in fault strength as much as possible. When assessing temporal changes in focal mechanisms, ideally the effects of stresses should be separated from those of fault strength (pore fluid pressures).

In this study, focal mechanism solutions were derived with a DC approximation because the magnitudes of events were low (*M*<1). In a volcanic environment, however, the moment tensors of VT events may have non-DC, isotropic or compensated-linear-vector-dipole (CLVD) components^46^,^47^. In general, the inner tensor product^48^ of a moment tensor and the regional stress tensor seems to be useful instead of the misfit angle to detect temporal changes in the local stress field (see Methods). Earthquakes release part of the stress field, and so the moment tensor of events must be somewhat similar to the stress tensor^23^. Thus, dissimilarities between the moment tensors and the regional stress tensor can be an indicator of temporal changes in the local stress field. The moving average of inner tensor products were calculated from DC moment tensors converted from focal mechanism solutions: the results indicated that the inner tensor product has a strong negative correlation with the misfit angle (Supplementary Figs 6a and 7a). Depressions of inner tensor products were detected in the precursory period, November 2014 and January to February 2015. Very similar results were obtained using possible non-DC moment tensors (Supplementary Figs 6c and 7b). This additional analysis showed that temporal stress changes can be robustly detected from the present data set although non-DC components were significant.

If seismic networks are improved, we could expect more earthquake focal mechanisms with better quality, which would provide higher resolution information on temporal changes in the local stress field and on the regional stress field. If we can monitor the temporal evolution of average misfit angles over a long period of time with multiple eruptive episodes at a particular volcano, the results would be useful for predicting eruptions by comparing current with previous behaviour. The minor eruption of Mt. Ontake in 2007 was undetected in real time but discovered afterwards by onsite investigation. By applying our approach to the data associated with such examples, this approach can be quantitatively evaluated.

The degree of temporal change in the local stress field depends on the magnitude of the stress perturbation caused by volcanic processes relative to the background stress level^1^,^2^,^3^,^4^,^17^. To create a practical warning system for volcanic eruptions, the absolute level of the local stress field (that is, rock strength) and/or the pressure threshold of magmatic/hydrothermal fluids required to trigger an eruption must be quantitatively evaluated, although differences in tectonic stresses, magma composition and local geology make it difficult to apply a single threshold to multiple volcanic systems. Nonetheless, the temporal stress changes observed using this approach can provide crucial constraints on the absolute stress level and the pressure threshold when combined with numerical modelling of the loading process and fluid pressurization. This is a promising method for understanding the signals that indicate an imminent eruption.

## Methods

### Seismicity and focal mechanism solutions

To examine the seismicity around Mt. Ontake, we visually re-picked the arrival times of P and S waves and the polarities of P waves for 11,057 events (May 2012 to March 2015) that were automatically detected by the WIN system^49^. We used these data to construct an earthquake catalogue for the Ontake region (Fig. 1a). Over 98% of the 475 events in the summit region have *M*<1. We also manually picked seismic events in the Ontake region by examining continuous waveforms in a 2-month window encompassing the eruption (30 August 2014 to 31 October 2014) to understand the changes in the number of seismic events at the summit region (Fig. 1c).

We estimated focal mechanism solutions from S:P amplitude ratios and P wave polarity data using the HASH software package after improving its code^21^. The improved software allows calculation of take-off angles taking into account the elevations of seismic stations. To calculate S:P amplitude ratios, we pre-processed velocity seismograms by integrating them to obtain the displacement and filtering with a bandpass window of 5–20 Hz. The parameters for determining focal mechanism solutions were as follows: polarity picks >8; maximum azimuthal gap <90°; maximum take-off angle gap <60° and signal-to-noise ratio >1.2. The fractions of reversed polarities to the final focal mechanism solutions were 19% on average. We obtained 94 relatively well-constrained focal mechanism solutions with RMS fault plane uncertainties of <45°, 53 of which possessed uncertainties of <35° (Supplementary Table 1).

### CMT data inversion analysis

The CMT data inversion method^23^ derives the stress pattern from a large amount of CMT data for earthquakes using Akaike's Bayesian information criterion^50^,^51^. We targeted the region around Mt. Ontake (latitude 35.62° N–36.16° N, longitude 137.3° E–137.9° E, depth 0–20 km) as the model region. We distributed 2,475 (15 × 15 × 11) tri-cubic B splines (basis functions) with equal spacing of 5 and 2.5 km local supports (grid intervals) in the horizontal and vertical directions, respectively, to represent the regional stress field. For the analysis, we used CMT data converted from focal mechanism solutions with RMS fault plane uncertainties of <35° (May 2012 to July 2014) using the well-known relationship between moment magnitude and seismic moment^52^. From the data set, we determined the best estimates of the model parameters (expansion coefficients of the basis functions) using the Yabuki–Matsu'ura inversion formula^53^. Each component of the stress tensor at any location was obtained as a superposition of the basis functions (Supplementary Fig. 3). Note that only the relative values of the six stress components are meaningful, not the absolute values. Estimation errors were obtained using an L2-norm of error tensors^25^. We also evaluated the variation of principal stress axes using a bootstrap method with 100 data sets in which each converted moment tensor was rotated around an arbitrary vector within uncertainties. The estimation errors in the principal stress axes were ∼15–20° and 5–10° in the summit and peripheral swarm regions, respectively (Fig. 1a).

### Evaluating misfit angles

For each focal mechanism solution, we calculated the theoretical slip vector (that is, the direction of the resolved shear traction on a nodal plane) using Cauchy's formula, given the regional stress pattern at the hypocentre. As the true fault plane, we selected the nodal plane with the smaller angular difference between the actual and theoretical slip vectors of both nodal planes and defined the angular difference as the misfit angle.

### Detecting temporal stress changes from moment tensors

The inner tensor product is a quantity used to measure the closeness of two tensors^48^ and ranges from −1 to 1, where +1 indicates that the two tensors are exactly the same (other than the scale factor), and −1 indicates that they are opposite. Before examining the effects of non-DC components of moment tensors to our conclusion, we first converted the focal mechanism solutions of 94 VT events (Supplementary Table 1) to DC moment tensors^23^, calculated the inner tensor products between the converted moment tensors and regional stress tensors, and evaluated the moving average of inner tensor products (Supplementary Figs 6a and 7a). For each event, we calculated a possible CLVD moment tensor, considering the axes of focal mechanism solutions and the P wave polarity data (Supplementary Fig. 6b). In the calculation, we assumed that the directions of the axial tensional and compressive dipoles were parallel to those of the T and P axes of events of reverse and normal faulting^22^, respectively. Otherwise, when the number of dilatational/compressive first-motion data was larger than that of compressive/dilatational data, we assumed that the CLVD moment tensors were calculated in the same manner as for reverse and normal faulting events, respectively. For remaining events, the CLVD moment tensors were obtained at random. We linearly combined these CLVD moment tensors with DC moment tensors to obtain possible non-DC moment tensors, where the weights of the CLVD moment tensors were assumed to be 30% of the DC moment tensors (Supplementary Fig. 6c). The possible moment tensors can explain the polarity data as well as the DC moment tensors (Supplementary Fig. 6a,c). Using these possible moment tensors, we evaluated the moving average of inner tensor products, indicating that effects of non-DC components were small for detecting temporal changes in the local stress field (Supplementary Fig. 7). The isotropic component of moment tensors does not influence the calculation of inner tensor products because the trace of the regional stress tensor inferred from focal mechanism solutions is zero.

## Additional information

**How to cite this article:** Terakawa, T. *et al.* Monitoring eruption activity using temporal stress changes at Mount Ontake volcano. *Nat. Commun.* 7:10797 doi: 10.1038/ncomms10797 (2016).

## Supplementary Material

Supplementary Information Supplementary Figures 1-7, Supplementary Table 1 and Supplementary References.
