# Supplementary material for: Monitoring eruption activity using temporal stress changes at Mount Ontake volcano
Source: Nat Commun. 2016 Feb 19;7:10797. doi: 10.1038/ncomms10797 (PMC4762890; doi:10.1038/ncomms10797)
Supplement: Supplementary Information — Supplementary Figures 1-7, Supplementary Table 1 and Supplementary References. [file ncomms10797-s1.pdf]

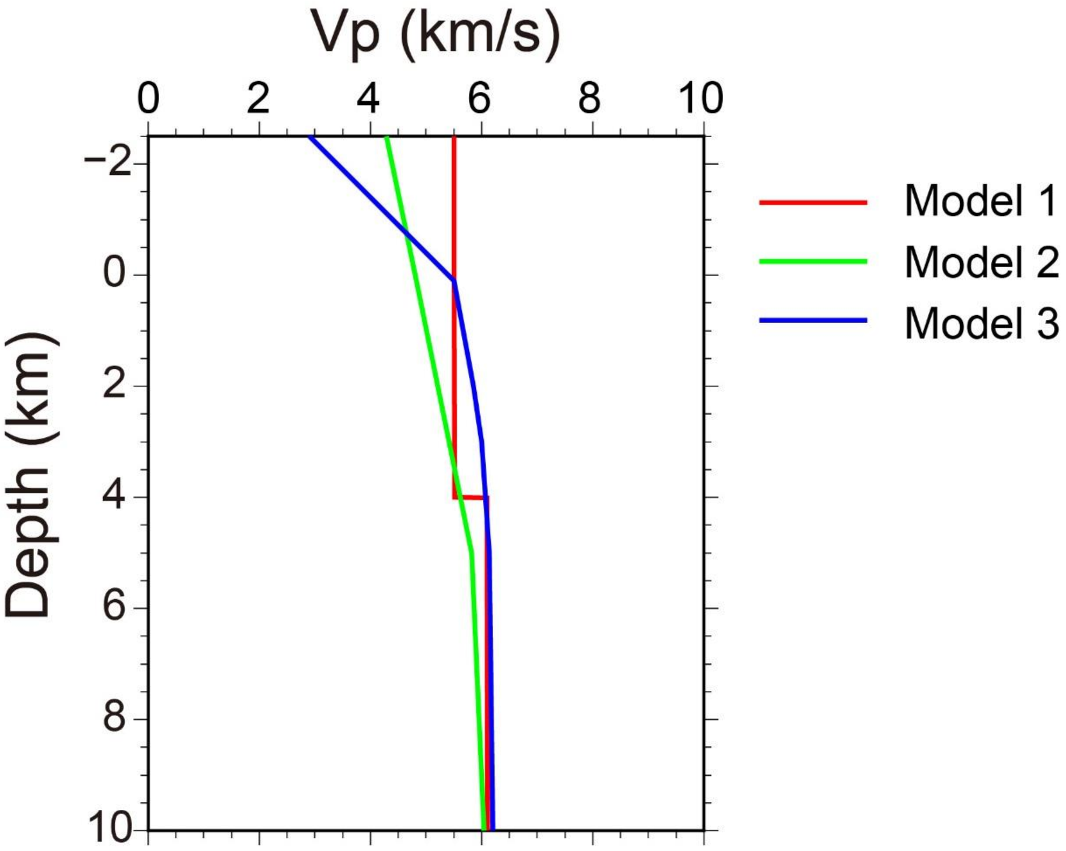

Supplementary Figure 1 One-dimensional P-Wave velocity structures. Model 1 is the Nagoya University routine model, Model 2 is the JMA 2001 model, and Model 3 is the Kato et al 2015 model[1]. We assumed a Poisson solid.

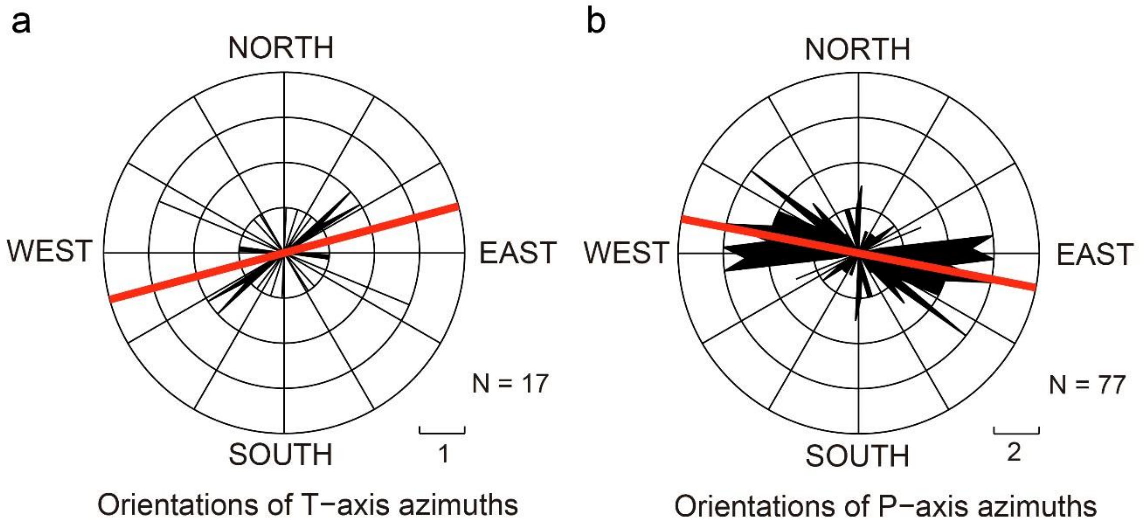

Supplementary Figure 2 Rose diagrams of T-axis and P-axis azimuths. (**a**) Rose diagram of the T-axis azimuths of events for the precursory period (31/08/2014-27/09/2014). The thick red line denotes the mean direction of the T-axes. (**b**) Rose diagram of the P-axis azimuths of events for the post-eruption period (27/09/2014-31/03/2015). The thick red line denotes the mean direction of the P-axes.

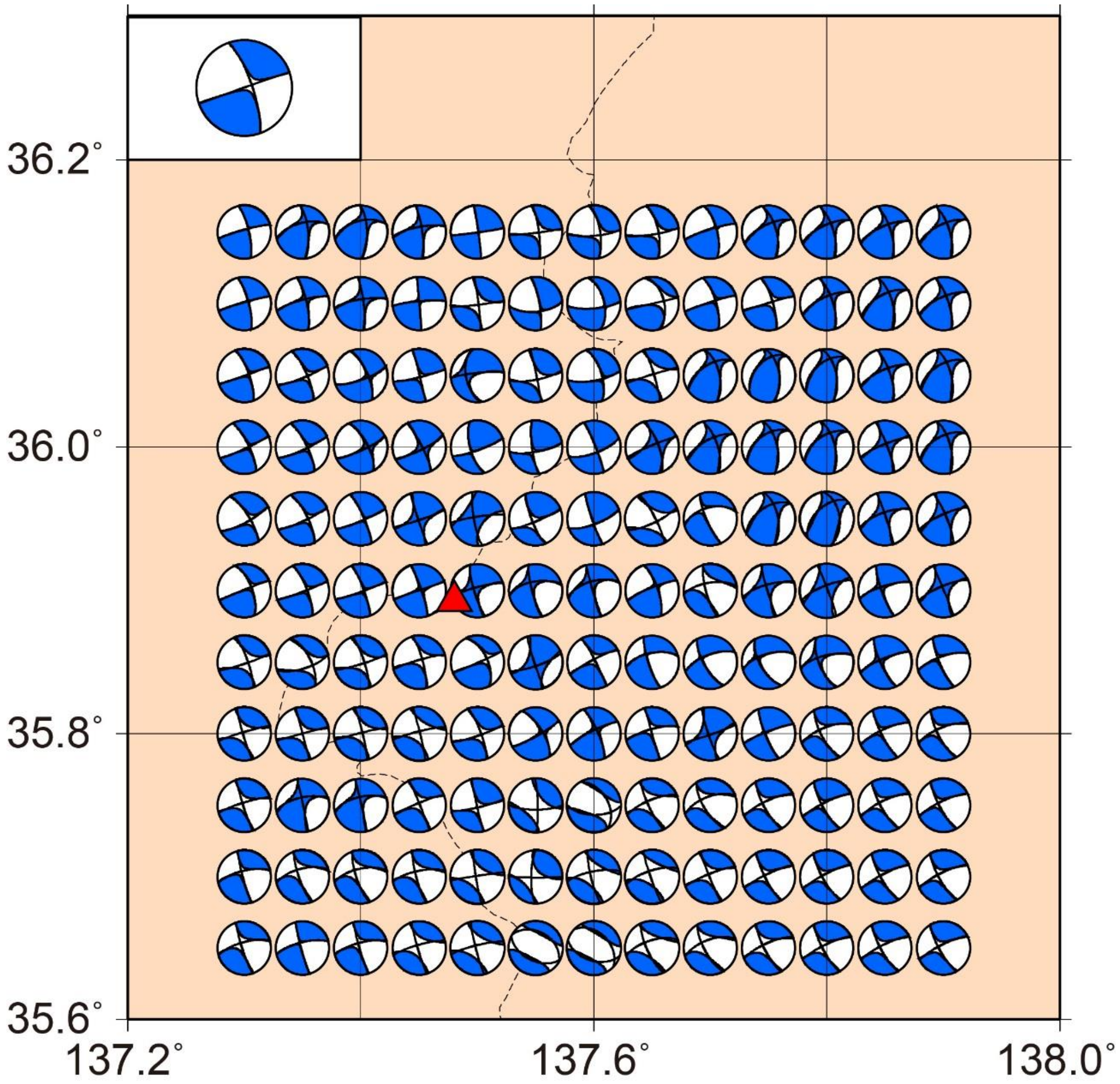

Supplementary Figure 3 Tectonic stress field around Mt. Ontake. The regional stress field in the Ontake region (depth = 5 km). The regional stress pattern is shown by lower hemisphere projection of focal spheres, where nodal planes are maximum shear planes. The inset shows the average stress pattern in the summit region (depth = 2 km). The red triangle shows Mt. Ontake. The black dashed line shows prefectural boundary between Nagano and Gifu prefectures.

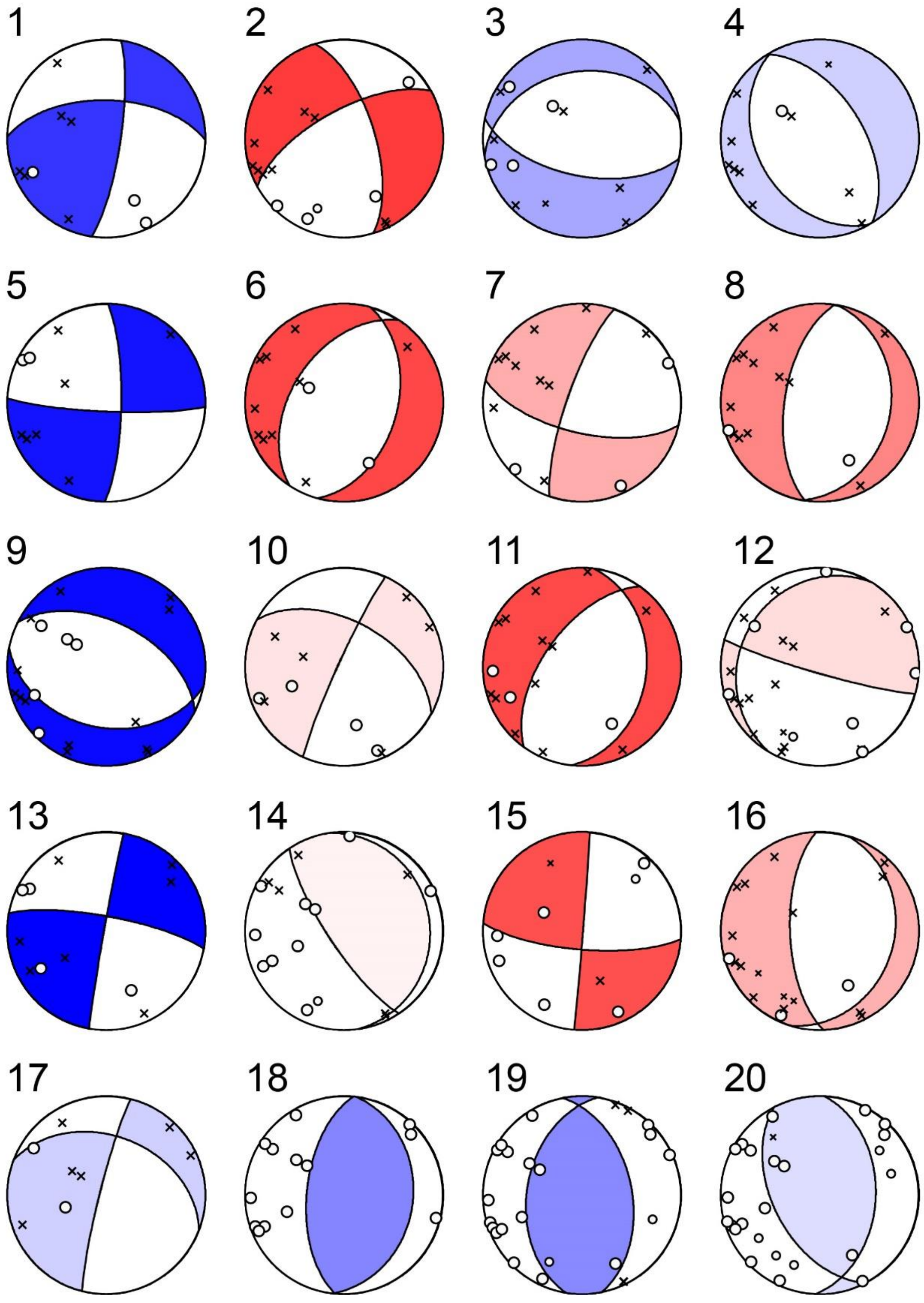

Supplementary Figure 4-1 Focal mechanism solutions of VT earthquakes. The focal mechanism solutions listed in Supplementary Table 1 are shown by lower hemisphere projection of focal spheres. The circles and crosses denote dilatational (downwards) and compressive (upwards) first motion. The colour of focal spheres indicates misfit angles between actual and theoretical slip vectors. The colour scales are the same as in Figs 2b and 2c.

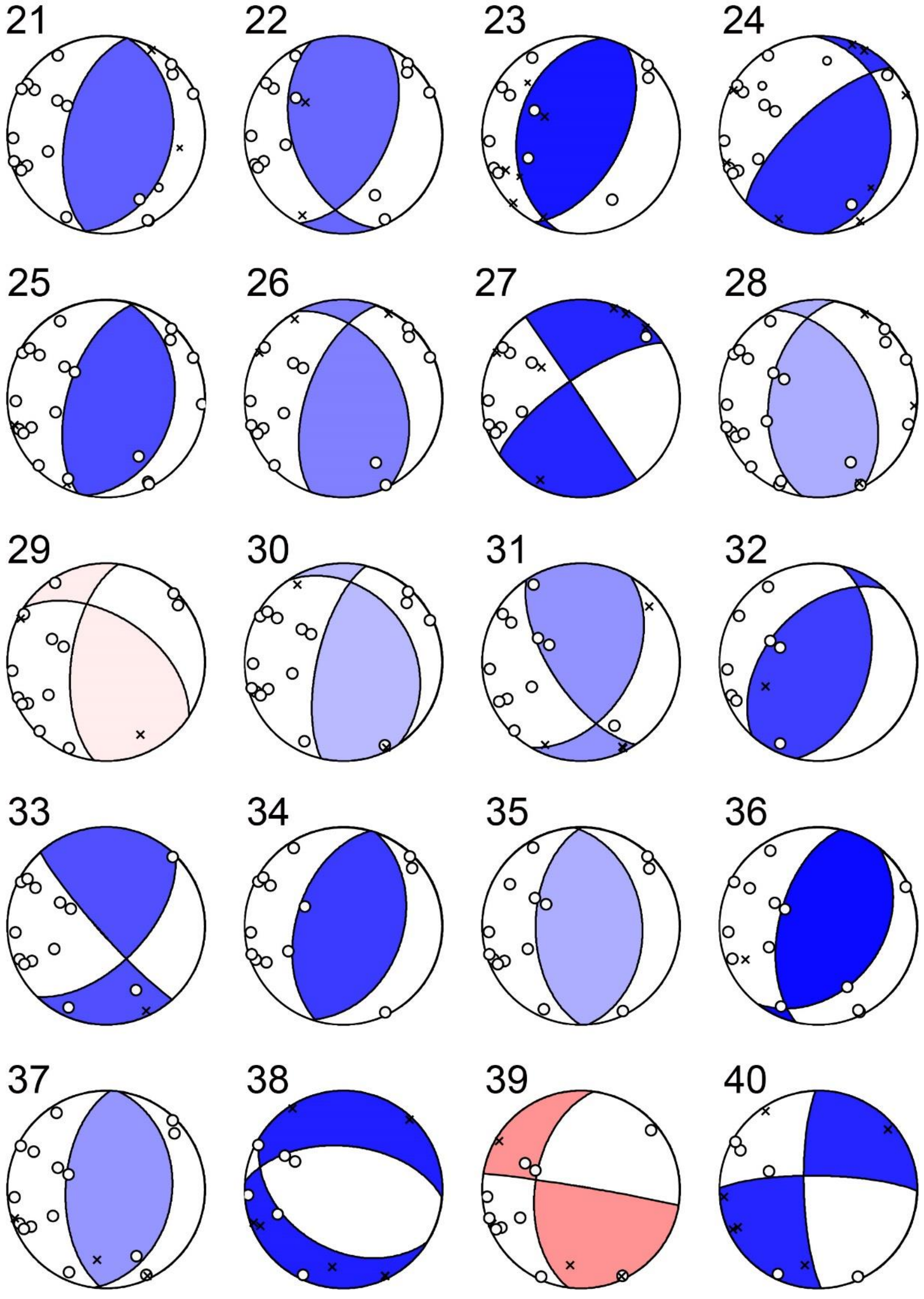

Supplementary Figure 4-2 Focal mechanism solutions of VT earthquakes.

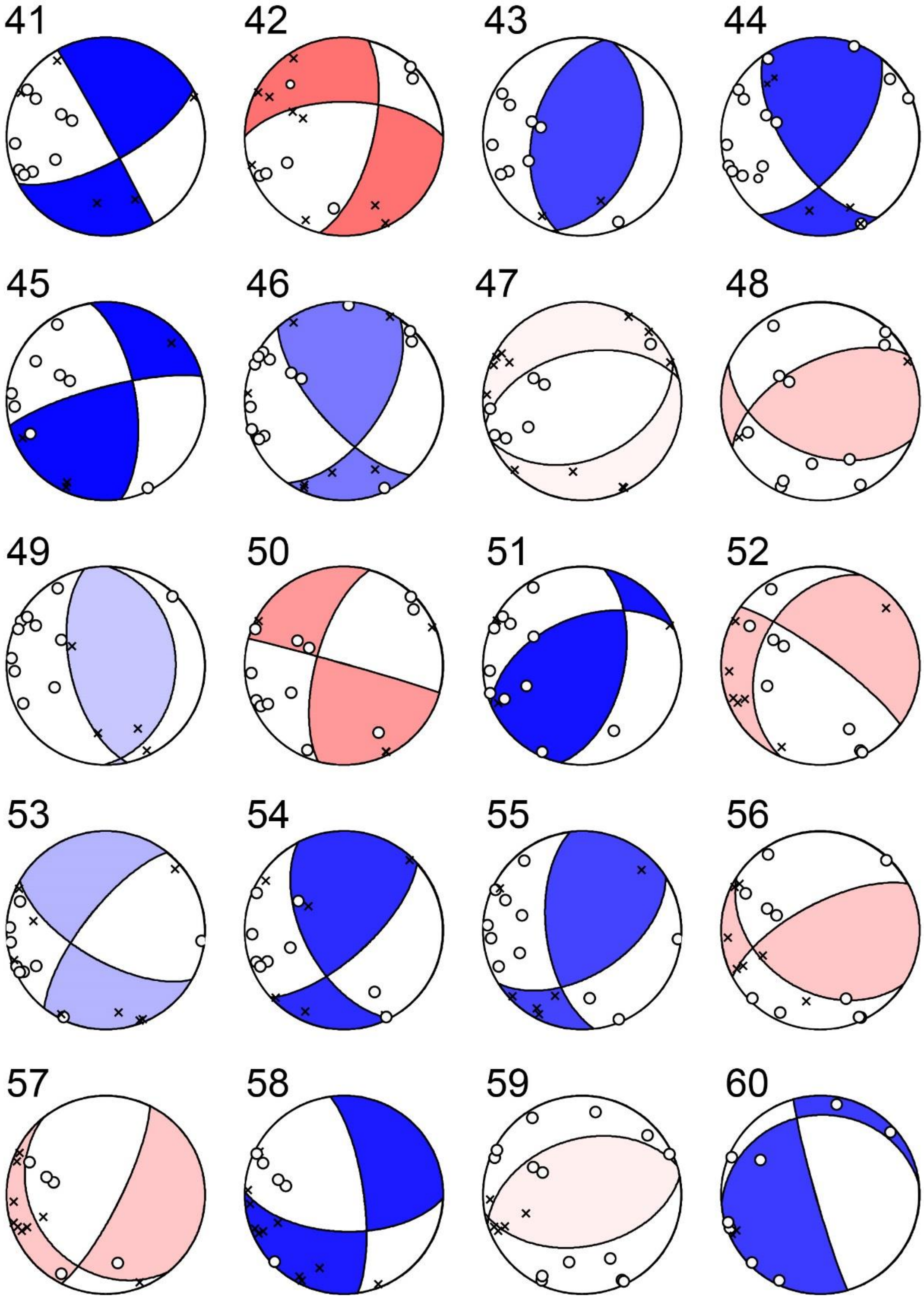

Supplementary Figure 4-3 Focal mechanism solutions of VT earthquakes.

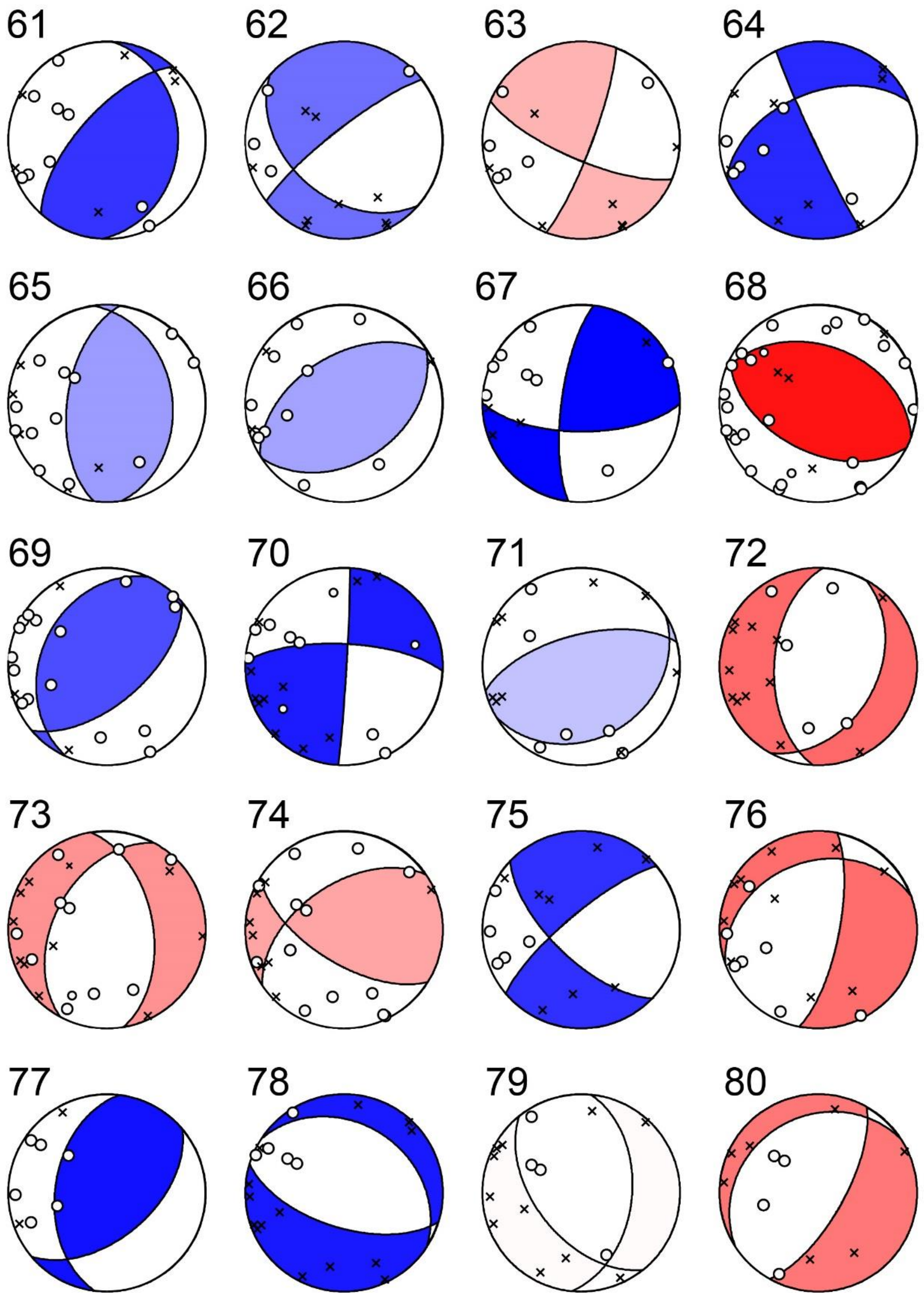

Supplementary Figure 4-4 Focal mechanism solutions of VT earthquakes.

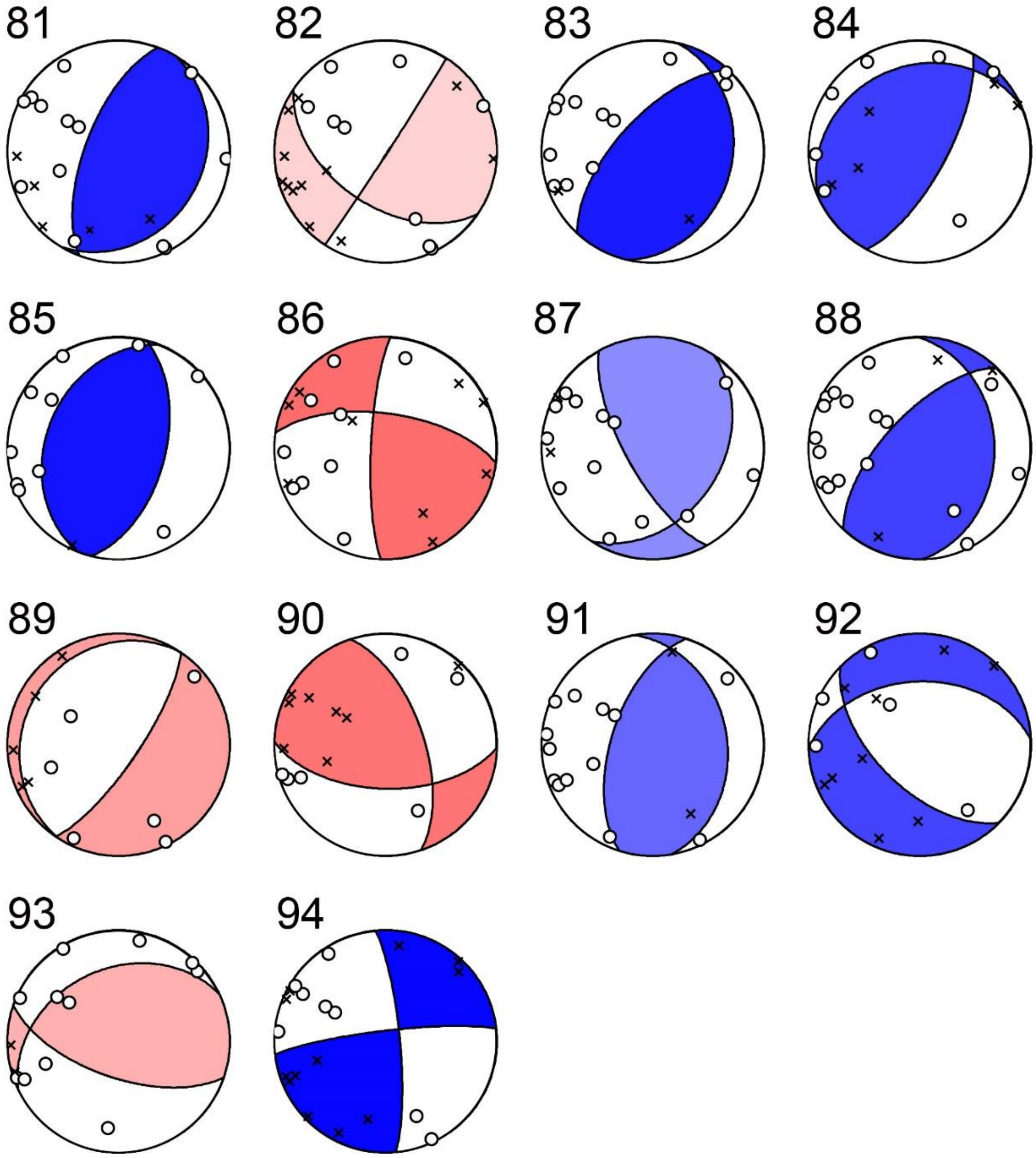

Supplementary Figure 4-5 Focal mechanism solutions of VT earthquakes.

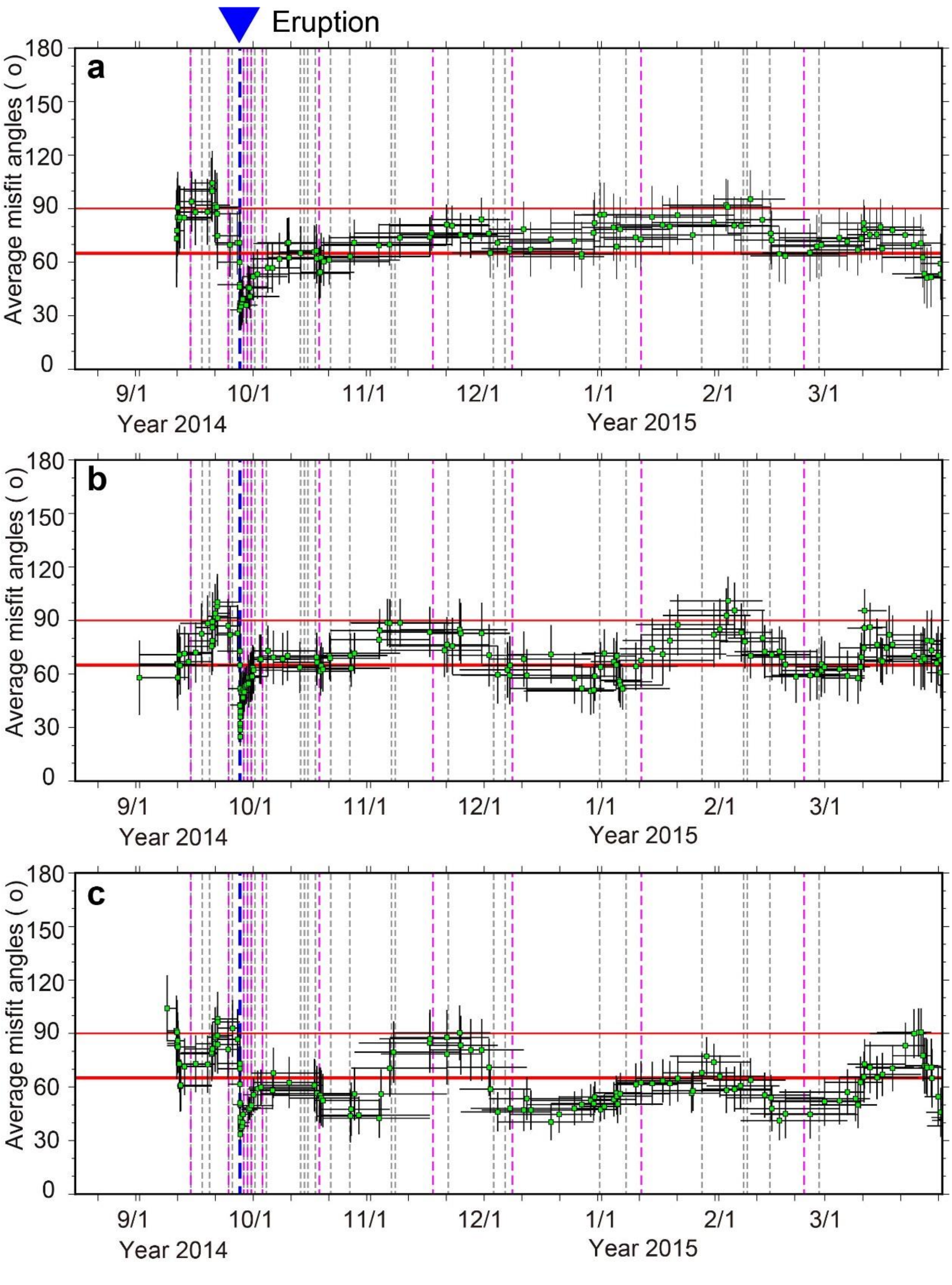

Supplementary Figure 5 Effects of velocity structures on temporal evolution of average misfit angles in the summit region. The moving averages of misfit angles of focal mechanism solutions obtained with Model 1-3 (Supplementary Fig. 1) are shown in (**a**)-(**c**), respectively. The light green squares, the black horizontal and vertical bars, the thick and thin red lines, and the blue, pink and grey dotted lines are the same as in Fig. 3a.

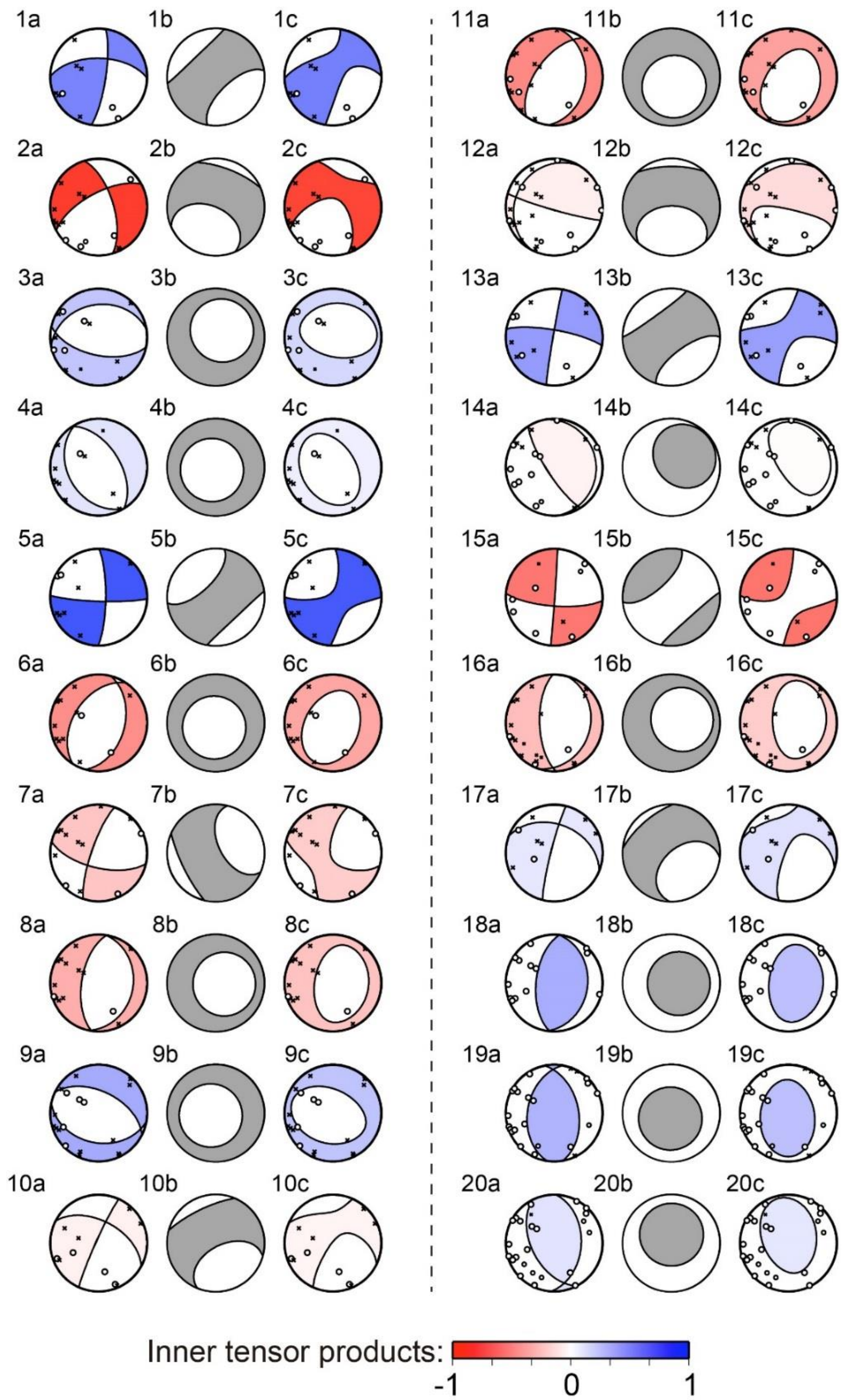

Supplementary Figure 6-1 DC moment tensors (focal mechanism solutions) and possible moment tensors of VT earthquakes. (**a**) The DC moment tensors of events listed in Supplementary Table 1 are shown by lower hemisphere projection of focal spheres. The circles and crosses denote dilatational (downwards) and compressive (upwards) first motion. The colour of focal spheres indicates inner tensor products between DC moment tensors and the regional stress tensors. (**b**) Assumed CLVD moment tensors of events. (**c**) Possible non DC moment tensors of events. The circles, crosses and colour of focal spheres are the same as in (**a**).

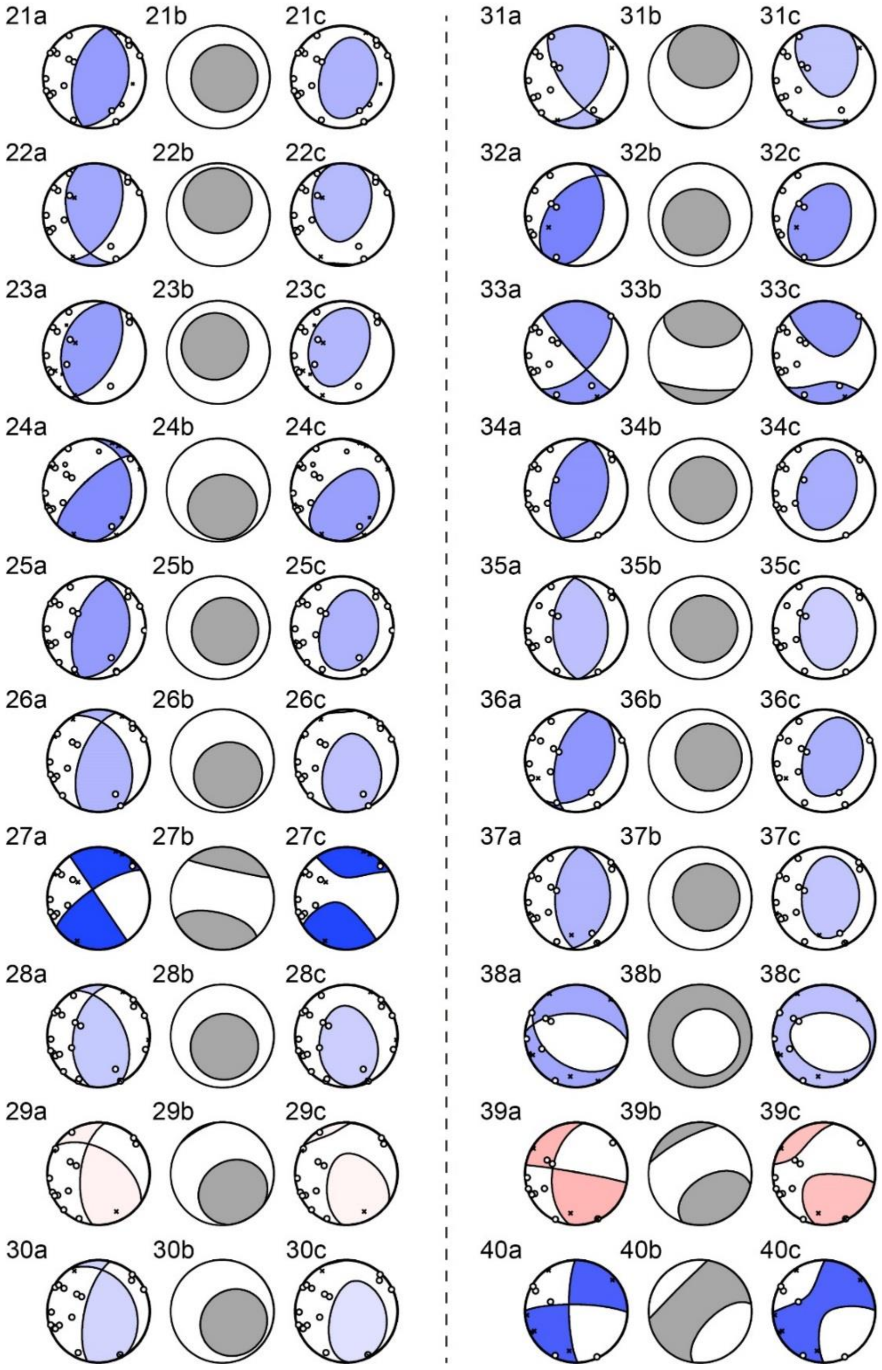

Supplementary Figure 6-2 DC moment tensors (focal mechanism solutions) and possible moment tensors of VT earthquakes.

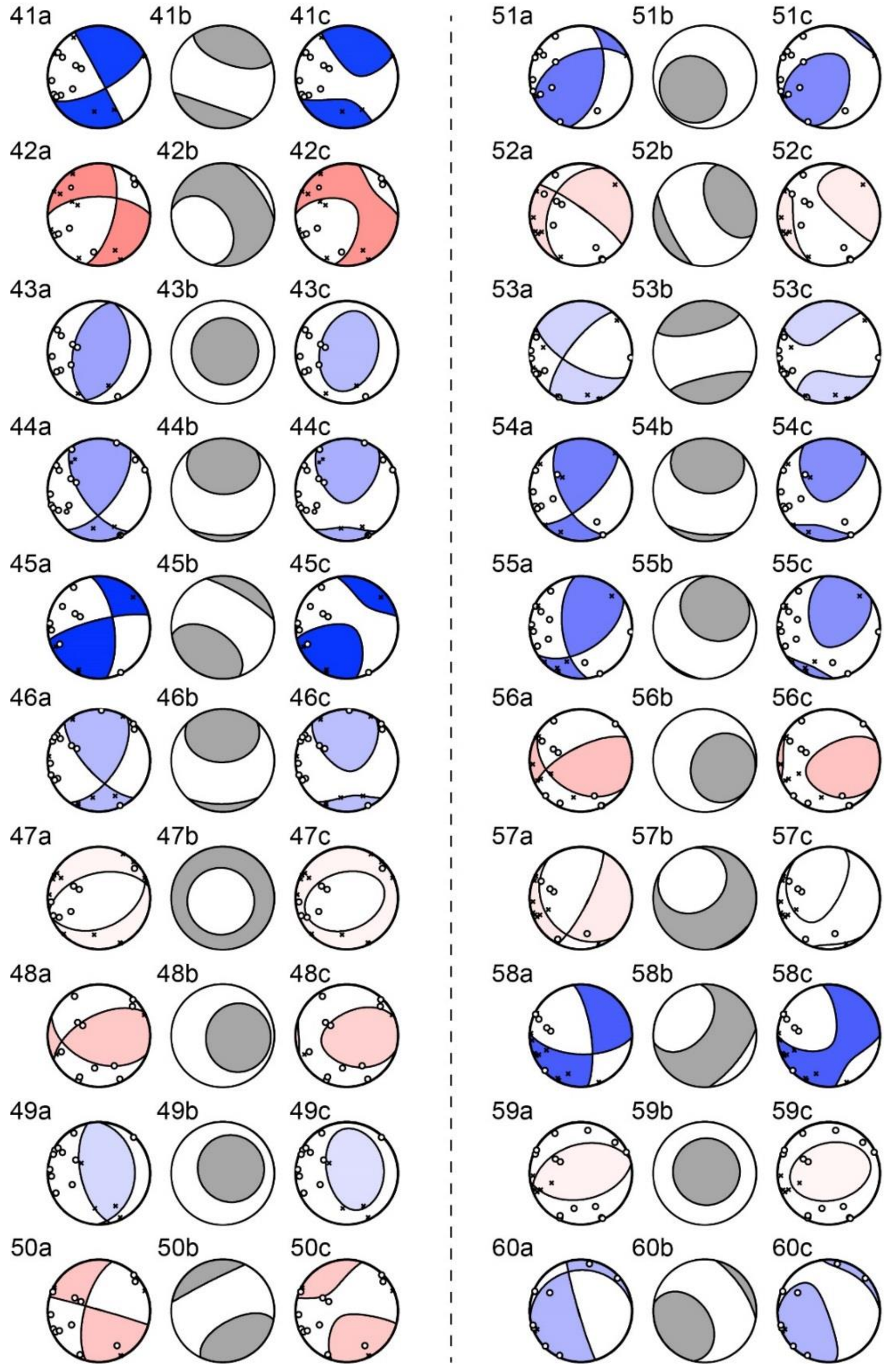

Supplementary Figure 6-3 DC moment tensors (focal mechanism solutions) and possible moment tensors of VT earthquakes.

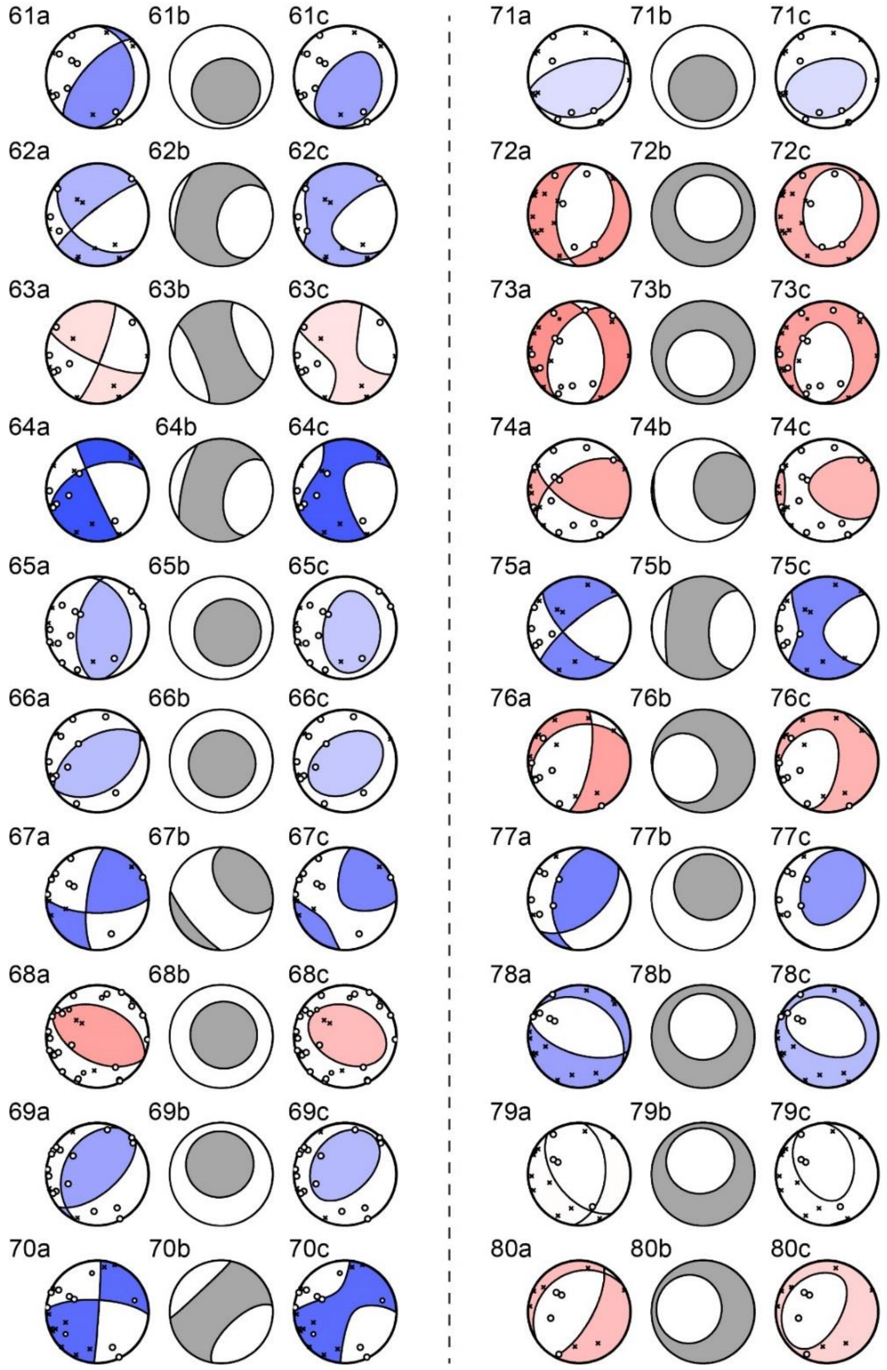

Supplementary Figure 6-4 DC moment tensors (focal mechanism solutions) and possible moment tensors of VT earthquakes.

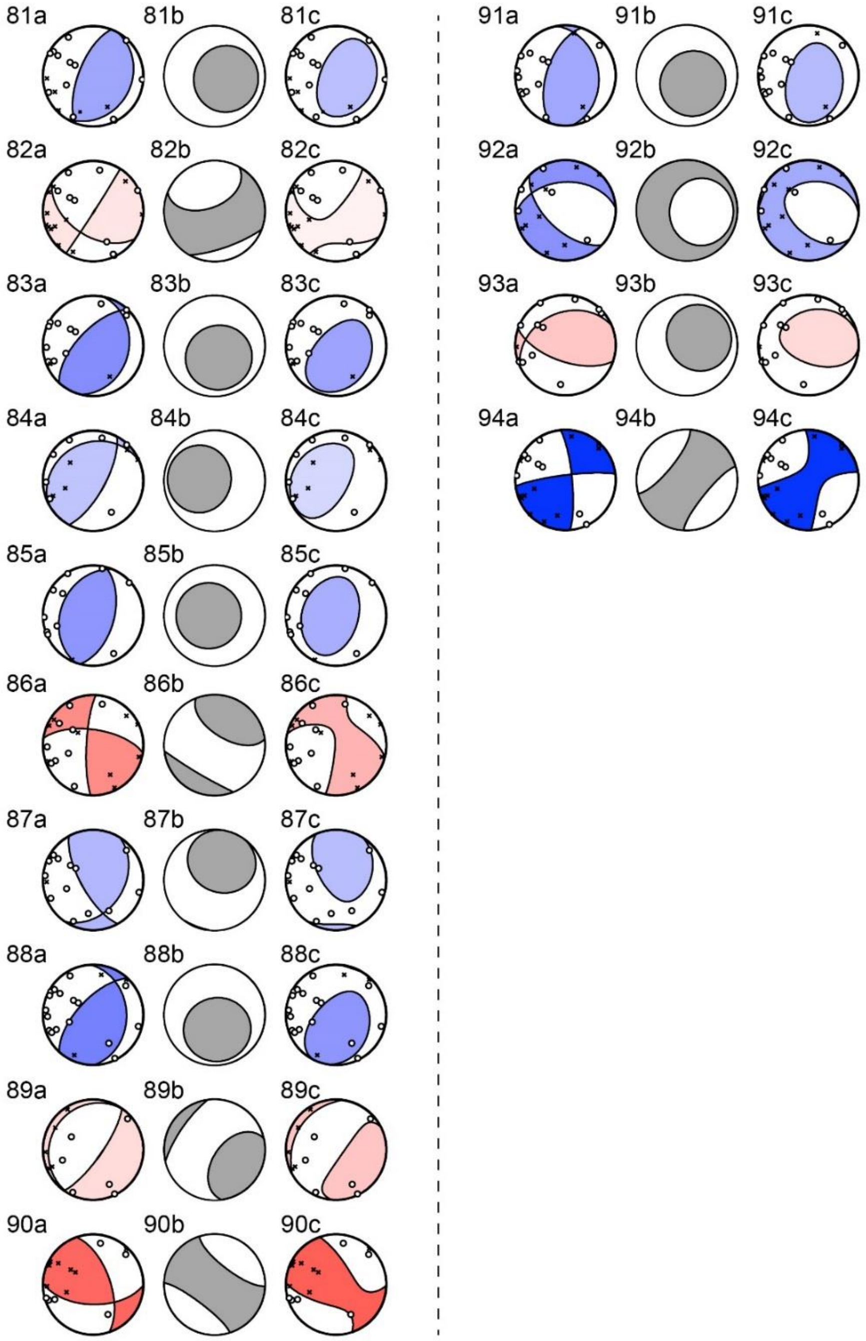

Supplementary Figure 6-5 DC moment tensors (focal mechanism solutions) and possible moment tensors of VT earthquakes.

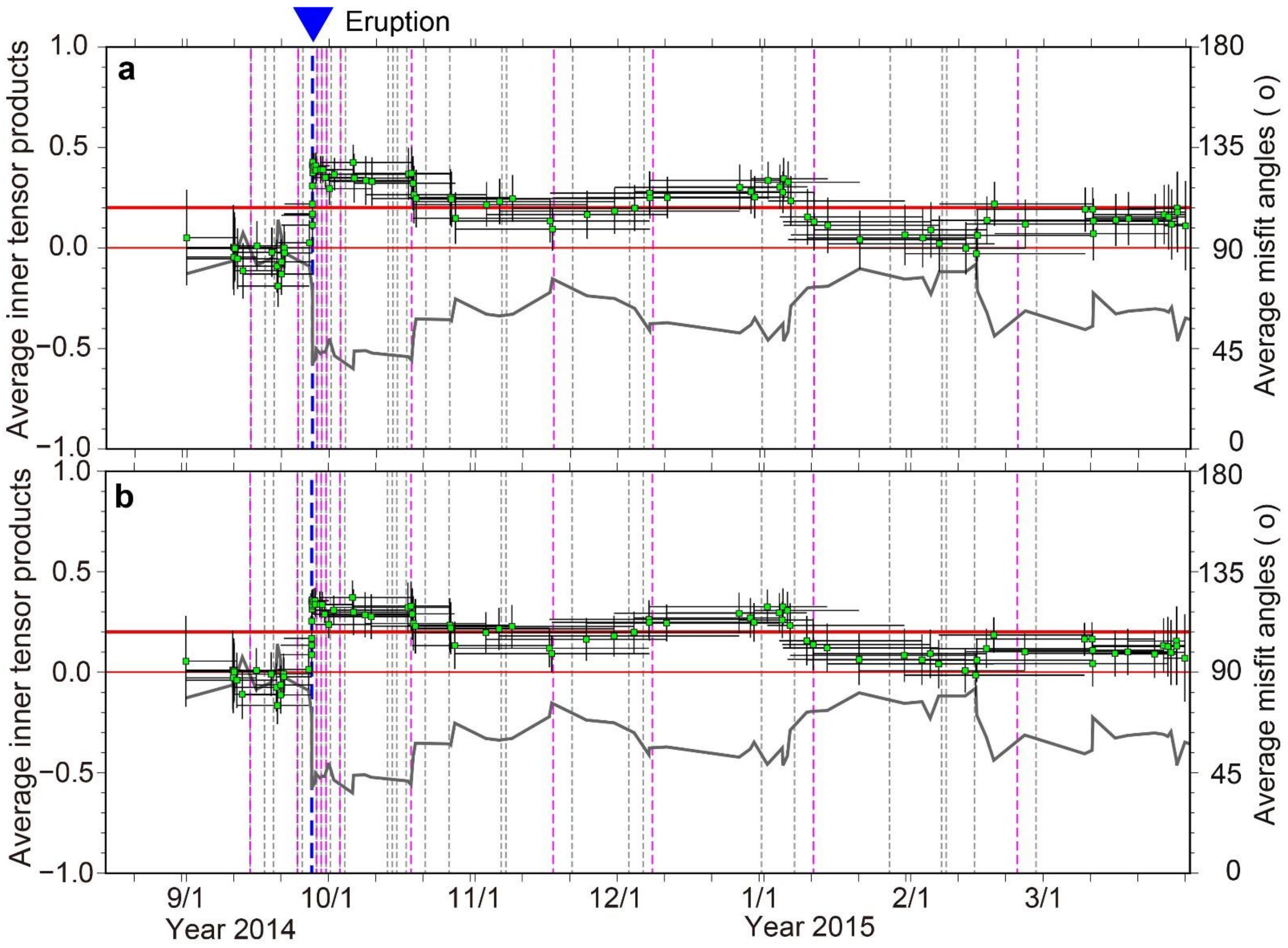

Supplementary Figure 7 Temporal evolution of average inner tensor products. (**a**) The moving average of inner tensor products between DC moment tensors of 94 VT events (Supplementary Table 1) and the regional stress tensors. The DC moment tensors were converted from focal mechanism solutions**.** (**b**) The moving average of inner tensor products between possible non DC moment tensors (Supplementary Fig. 6c) and the regional stress tensors. The black horizontal bars denote the time window for calculating average inner tensor products, and vertical bars denote the standard errors. The thick red lines show the threshold value of inner tensor products (0.2), which corresponds to the threshold misfit angle (65°). The thin red lines show another threshold value corresponding to the misfit angle of 90°. The grey lines show the moving average of misfit angles in Fig. 3a. The blue, pink and grey dotted lines are the same as in Fig. 3.

Supplementary Table 1 List of VT earthquakes

Evn: Event number in Supplementary Figure 4. Rank: Focal mechanism quality. A, B, and C denote those with RMS fault plane uncertainties $\leq 25^{o}$, $\leq 35^{o}$, and $\leq 45^{o}$, respectively. The hypocenters were obtained using the velocity structure, Model 1 (Supplementary Fig. 1). The parameters of focal mechanisms were shown by one of the fault plane solutions.

Evn	Date	Time (hr:min, sec)		Lat.	Lon.	Depth (km)	Mag.	Str	Dip	Rake	Rank
1	31/8/2014	23:25	10.46	35.88483	137.4738	1.19	-0.5	271	58	166	C
2	10/9/2014	20:26	42.22	35.88733	137.4767	1.37	-0.1	343	65	-154	B
3	10/9/2014	22:22	13.00	35.89167	137.4825	1.69	-0.3	260	33	-109	C
4	11/9/2014	2:27	46.2	35.889	137.4755	1.71	-0.1	328	54	-91	B
5	11/9/2014	3:01	37.89	35.88433	137.4723	1.83	-0.4	92	83	-168	C
6	11/9/2014	17:06	43.99	35.8845	137.472	1.49	-0.3	15	45	-103	B
7	12/9/2014	21:06	6.39	35.88617	137.472	1.76	-0.3	199	78	-154	C
8	15/9/2014	20:07	26.94	35.88717	137.4752	1.47	-0.4	10	29	-89	B
9	18/9/2014	23:46	4.46	35.88883	137.475	1.59	-0.5	101	38	-102	B
10	20/9/2014	0:45	28.99	35.88283	137.4628	2.09	-0.1	298	52	-174	C
11	20/9/2014	6:00	27.32	35.88733	137.4757	1.48	-0.2	6	35	-115	B
12	21/9/2014	0:19	52.38	35.88717	137.4783	1.46	0	106	85	109	B
13	21/9/2014	0:19	58.35	35.87883	137.4753	2.08	0	280	78	-178	B
14	21/9/2014	12:54	35.94	35.88817	137.476	1.56	0.4	352	14	114	B
15	21/9/2014	14:57	12.91	35.88617	137.4688	2.75	0.5	4	89	165	B
16	26/9/2014	21:01	46.6	35.887	137.476	1.88	0.4	17	26	-73	B
17	27/9/2014	11:46	5.41	35.88883	137.475	2.4	0.1	291	40	-171	C
18	27/9/2014	12:03	36.44	35.88917	137.4757	0.5	0.9	7	31	91	B
19	27/9/2014	12:30	49.08	35.88667	137.4743	0.28	0.4	349	48	76	B
20	27/9/2014	12:37	50.17	35.89017	137.4743	0.86	1.1	153	51	70	B
21	27/9/2014	12:41	5.70	35.889	137.4742	0.51	0.8	13	34	90	B
22	27/9/2014	12:51	5.42	35.89183	137.4727	0.72	0.4	161	47	55	C
23	27/9/2014	13:42	41.64	35.88467	137.4752	0.82	0.3	28	55	99	B
24	27/9/2014	13:45	35.50	35.88567	137.4745	0.21	0.4	227	71	111	B
25	27/9/2014	13:57	41.51	35.88983	137.4757	1.13	0.4	11	32	85	B
26	27/9/2014	14:17	17.27	35.88983	137.4732	0.47	0.7	200	60	119	B
27	27/9/2014	14:42	47.69	35.8905	137.4732	0.55	0.2	236	73	180	C
28	27/9/2014	21:49	45.74	35.88517	137.4762	0.86	1	190	49	116	B
29	28/9/2014	5:59	56.84	35.882	137.4767	0.1	-0.2	187	61	135	B
30	28/9/2014	7:44	41.15	35.89083	137.4745	1.2	0.9	193	68	111	B
31	29/9/2014	6:04	44.97	35.88417	137.4778	0.88	0.3	30	46	144	C

32	29/9/2014	17:56	21.18	35.8835	137.473	0.99	0	222	47	111	C
33	30/9/2014	5:13	38.27	35.8845	137.4747	0.97	0	44	59	172	C
34	1/10/2014	2:53	31.97	35.8845	137.4753	0.55	0.2	18	40	92	C
35	2/10/2014	5:05	23.66	35.88567	137.4767	0.65	0.5	355	38	86	B
36	6/10/2014	3:29	38.36	35.885	137.4747	1.25	0	38	35	110	B
37	6/10/2014	8:50	7.92	35.88317	137.4748	0.76	1	182	56	87	C
38	8/10/2014	19:07	21.43	35.88617	137.4742	-0.2	-0.3	275	54	-107	C
39	10/10/2014	1:49	30.78	35.88367	137.474	0.13	-0.2	188	51	177	C
40	17/10/2014	20:43	5.80	35.8835	137.4718	0.09	-0.1	271	79	-168	C
41	18/10/2014	11:36	23.97	35.88417	137.475	1.12	0.3	62	69	-179	C
42	18/10/2014	18:39	55.43	35.884	137.4743	0.51	0.1	14	66	-148	B
43	19/10/2014	0:17	57.4	35.88133	137.4673	1.42	-0.4	18	42	94	C
44	19/10/2014	10:01	56.72	35.88217	137.4737	0.4	0.4	38	62	147	A
45	26/10/2014	15:52	38.38	35.88633	137.4738	1.01	0.1	256	78	155	C
46	26/10/2014	22:06	10.32	35.88583	137.4733	0.43	0.2	34	59	155	C
47	27/10/2014	17:38	24.60	35.88367	137.475	0.69	0.4	66	41	-98	C
48	3/11/2014	6:37	22.73	35.88683	137.475	1.45	-0.1	112	45	130	B
49	6/11/2014	2:01	54.90	35.88567	137.476	0.96	0.1	1	30	102	B
50	8/11/2014	19:38	37.17	35.88383	137.4778	0.59	-0.1	195	67	178	C
51	16/11/2014	16:18	54.00	35.88667	137.4743	0.69	0.2	247	50	132	C
52	17/11/2014	4:36	36.07	35.88583	137.4737	0.99	0.3	306	81	-126	B
53	24/11/2014	13:29	47.37	35.87683	137.4613	-0.13	0.4	218	72	-154	C
54	30/11/2014	8:28	33.43	35.88783	137.4743	0.95	0.5	47	68	144	C
55	4/12/2014	13:58	17.36	35.88167	137.4577	1.15	0.3	57	53	141	B
56	7/12/2014	17:34	22.48	35.88334	137.471	0.71	0.4	123	38	144	A
57	7/12/2014	20:31	42.72	35.88283	137.4608	0.98	0.1	141	27	-153	C
58	11/12/2014	11:48	21.51	35.88033	137.4617	0.4	0.4	92	60	-161	C
59	26/12/2014	18:17	50.19	35.887	137.472	0.87	0.7	253	43	85	A
60	29/12/2014	3:21	26.62	35.88667	137.4738	-0.25	-0.3	264	21	-169	B
61	29/12/2014	23:13	13.13	35.88683	137.476	0.39	0	222	67	108	C
62	1/1/2015	17:26	29.55	35.88817	137.478	1.23	0.2	232	81	-131	B
63	4/1/2015	6:40	31.26	35.88583	137.4752	0.8	-0.4	21	81	164	B
64	4/1/2015	22:15	0.77	35.89183	137.4747	0.83	-0.6	248	50	-176	A
65	5/1/2015	0:22	31.09	35.889	137.4757	1.06	-0.4	188	57	99	C
66	5/1/2015	23:12	43.77	35.89117	137.475	0.6	-0.1	238	50	92	C
67	6/1/2015	12:27	22.41	35.88683	137.4702	0.55	0.2	90	67	162	C
68	10/1/2015	1:19	50.18	35.8885	137.4777	1.02	0.8	117	49	94	B
69	11/1/2015	10:19	53.29	35.8885	137.4775	0.4	0	205	33	69	B

70	14/1/2015	9:47	54.47	35.88667	137.4715	0.44	0.6	273	71	178	A
71	21/1/2015	2:38	5.18	35.88817	137.4708	0.68	0.5	256	64	98	C
72	30/1/2015	16:26	54.16	35.88783	137.4743	1.46	0.4	183	53	-106	B
73	3/2/2015	10:31	10.03	35.88766	137.4713	1.2	0.6	350	51	-116	C
74	5/2/2015	3:00	0.23	35.88583	137.473	0.96	0.5	122	62	136	A
75	6/2/2015	21:51	43.32	35.891	137.476	0.94	-0.3	230	77	-156	C
76	12/2/2015	12:38	18.62	35.888	137.4768	0.84	0.1	11	73	-116	C
77	14/2/2015	18:43	27.08	35.89083	137.48	0.78	-0.4	50	52	119	C
78	14/2/2015	23:15	55.35	35.888	137.4722	0.08	0.8	107	61	-98	B
79	16/2/2015	23:59	3.27	35.88783	137.47	0.98	0.4	141	52	-121	B
80	18/2/2015	13:30	2.74	35.89117	137.4762	0.71	-0.1	29	69	-101	C
81	25/2/2015	2:03	47.39	35.88733	137.473	1.17	-0.3	201	66	86	B
82	9/3/2015	17:53	50.89	35.88733	137.472	1.23	0.6	124	49	-178	B
83	11/3/2015	7:39	55.28	35.8895	137.475	0.9	0.2	13	29	63	B
84	11/3/2015	10:29	13.84	35.89	137.4762	0.66	-0.1	246	24	125	B
85	11/3/2015	10:52	51.34	35.88917	137.4752	-0.54	0.2	202	35	96	C
86	16/3/2015	4:41	38.90	35.8885	137.4772	1.17	-0.4	183	79	154	B
87	18/3/2015	20:30	13.70	35.88783	137.4745	1.07	0.2	33	38	146	B
88	24/3/2015	12:46	26.23	35.889	137.476	1.48	0.8	223	65	113	B
89	26/3/2015	9:24	59.80	35.887	137.4733	0.38	-0.1	213	14	-92	B
90	27/3/2015	7:24	12.47	35.88867	137.4733	1.19	-0.1	341	62	142	B
91	28/3/2015	0:07	24.52	35.88833	137.4762	0.81	-0.2	198	59	105	C
92	29/3/2015	3:14	27.37	35.89167	137.4812	0.56	-0.2	134	58	-60	B
93	29/3/2015	5:01	52.39	35.88883	137.476	-0.2	-0.5	109	63	113	C
94	30/3/2015	22:00	13.97	35.888	137.4703	0.53	0.4	263	82	169	B

References

1. Kato, A. *et al.* Preparatory and precursory processes leading up to the 2014 phreatic eruption of Mount Ontake, Japan. *Earth, Planets Space* **67**, 111 (2015).
